# Serum Markers of Refractoriness and Enteropathy-Associated T-Cell Lymphoma in Coeliac Disease

**DOI:** 10.3390/cancers13102289

**Published:** 2021-05-11

**Authors:** Marco Vincenzo Lenti, Nicola Aronico, Paolo Giuffrida, Valentina Antoci, Giovanni Santacroce, Alessandro Vanoli, Catherine Klersy, Gino Roberto Corazza, Antonio Di Sabatino

**Affiliations:** 1 Department of Internal Medicine, IRCCS San Matteo Hospital Foundation, University of Pavia, 27100 Pavia, Italy; marco.lenti@unipv.it (M.V.L.); n.aronico@smatteo.pv.it (N.A.); paolo.giuffrida01@universitadipavia.it (P.G.); valentina.antoci01@universitadipavia.it (V.A.); giovanni.santacroce01@universitadipavia.it (G.S.); gr.corazza@smatteo.pv.it (G.R.C.); 2 Anatomic Pathology Unit, IRCCS San Matteo Hospital Foundation, Department of Molecular Medicine, University of Pavia, 27100 Pavia, Italy; alessandro.vanoli@unipv.it; 3 Clinical Epidemiology and Biometry, IRCCS San Matteo Hospital Foundation, University of Pavia, 27100 Pavia, Italy; c.klersy@smatteo.pv.it

**Keywords:** β2-microglobuline, chromogranin A, refractory coeliac disease, serum markers

## Abstract

**Simple Summary:**

Coeliac disease is a common chronic enteropathy that may lead to severe complications, including refractoriness (i.e., nonresponsiveness to a gluten-free diet) and enteropathy-associated T-cell lymphoma. In this study, we found that two serum markers, namely chromogranin A and β2-microglobuline, can predict these complications in patients with coeliac disease.

**Abstract:**

The persistence or recurrence of symptoms in patients with coeliac disease (CD), despite a gluten-free diet (GFD), must prompt further work-up for excluding refractory CD (RCD). The aim of this study was to assess the accuracy of serum markers in predicting refractoriness in CD patients. This study included 72 patients affected by CD followed-up at our center, namely 49 uncomplicated CD before and after GFD and 23 RCD. Serum levels of chromogranin A (CgA) and β2-microglobuline were measured at baseline and at follow-up (median time of 13 months) in each group of patients. Cut-off points for each marker were estimated to differentiate RCD from uncomplicated CD patients. Serum levels of CgA and β2-microglobuline were significantly higher in patients with RCD compared to uncomplicated CD (*p* < 0.001), both at baseline and at follow-up, with no significant difference between RCD type 1 and type 2. The estimated cut-off point for CgA was 90.2 ng/mL (sensitivity 83%, specificity 100%), while for β2-microglobuline it was 696 mcg/L (sensitivity 100%, specificity of 100%). To conclude, CgA and β2-microglobuline could be useful serological markers of refractoriness in CD, with the ability to discriminate those patients who should undergo upper gastrointestinal endoscopy for making a definite diagnosis.

## 1. Introduction

Coeliac disease (CD) is a chronic immune-mediated intolerance to gluten proteins that causes villous flattening of the small bowel mucosa in genetically susceptible individuals [1,2]. The pathogenesis of CD is multi-factorial and involves immunologic, genetic, and environmental factors that interact in causing this disorder [2].

To date, epidemiological data suggest a prevalence of CD in the general population of 1 case per 130–140 individuals, with an apparent lower frequency in Asian regions [3,4,5,6]. Almost all CD patients carry HLA-DQ2, HLA-DQ8, or their variants, however, up to 40% of European and Asian people also carry these genes, indicating that other markers are necessary to support CD diagnosis in dubious cases [7].

Currently, the only effective available treatment for CD is a life-long, strict, gluten-free diet (GFD), which is able to restore small bowel mucosa integrity in most cases, ameliorating most of CD-related clinical manifestations [8,9], and preventing possible complications, some of them potentially life-threatening, such as refractory CD (RCD), ulcerative jejunoileitis, and enteropathy-associated T-cell lymphoma (EATL) [10].

RCD is characterized by the absence of clinical and histological (i.e., recovery of villous atrophy) response after at least 12 months of a strict GFD, in the absence of overt malignancy [11]. RCD is clinically characterized by a malabsorption syndrome which poses a challenge in clinical management [11]. An estimated annual incidence of 0.83 per 10,000 patients has been reported. Novel therapies for the treatment of RCD are currently under investigation [12].

RCD can be further classified as primary or, more commonly, secondary, i.e., the initial response to a GFD, followed by a relapse [13], or as type 1 and type 2, mainly on the basis of the number of aberrant intraepithelial lymphocytes (IELs) identified by flow cytometry and on the basis of the presence or absence of T-cell receptor rearrangements [11]. RCD type 2 is a precursor of a rare type of lymphoma known as EATL [14]. Currently, there are no serum markers able to predict refractoriness in CD, as CD antibodies are usually negative in this condition [11], and other markers of enterocyte loss failed to prove any usefulness in this setting. For example, serum levels of citrulline, a nonprotein amino acid reflecting the enterocyte mass, were not able to discriminate between the presence or absence of villous atrophy [15]. Hence, that of RCD is a diagnosis of exclusion, primarily based on clinical grounds and duodenal biopsy confirmation, making sure that the patient is on a GFD through a validated questionnaire/interview, as no validated biological markers of adherence to a GFD exist in clinical practice [16]. In light of the increasingly pressing need to make an early and correct diagnosis of RCD [9], the goal of our study was to evaluate a number of serum biomarkers in predicting a diagnosis of RCD in a clinical setting.

## 2. Materials and Methods

### 2.1. Patients

In this single-center, prospective, proof-of-concept, observational study, we collected data from adult patients affected by CD (*n* = 72, 44 females). Among them, 23 were affected by RCD (16 RCD type 1, mean age 68 ± 13 years, and 7 RCD type 2, mean age 66 ± 12 years) and 49 by uncomplicated CD (mean age 35 ± 12 years). All these patients were evaluated at baseline and at follow-up (median time of 13 months). The baseline for uncomplicated CD was the time of diagnosis of CD, when these patients had not yet followed a GFD. the follow-up for uncomplicated CD patients was made at least after 12 months of a GFD. Assessment of adherence to a GFD was made through a validated score [16]. Moreover, all patients enrolled had been instructed on how to avoid possible gluten-contaminated food by a specialist dietitian.

### 2.2. Clinical Features

Diagnosis of uncomplicated CD at baseline, according to internationally agreed guidelines [17], was based on the positivity of serum anti-endomysial and/or anti-tissue transglutaminase IgA antibodies associated with typical histopathological lesions, namely villous atrophy, increased intraepithelial lymphocyte infiltration, and crypt hyperplasia, before starting a GFD. None of the included patients had either IgA deficiency or common variable immune deficiency. RCD was diagnosed on the basis of the absence of clinical and histological response after 12 months of a strict GFD, in the absence of other causes of nonresponsiveness to a GFD or overt malignancy [11]. We classified RCD into type 1 and type 2 on the basis of the number of aberrant IELs identified by flow cytometry. Patients with less than 20% of aberrant IELs were classified as having RCD type 1, while patients with 20% or more aberrant IELs were classified as having RCD type 2 [11]. The median duration of disease, calculated at follow-up, was 1 year for uncomplicated CD patients, 3 years (range 1–14 years) for RCD type 1 patients, and 12 years (range 1–15 years) for RCD type 2 patients. At last follow-up (median of 13 months for all groups), patients with uncomplicated CD, who had followed a strict GFD for at least 12 months, referred the absence or a marked improvement of abdominal pain, diarrhoea, or other clinical presentations. On the contrary, patients with RCD reported the persistence of symptoms, in particular a malabsorption syndrome, with diarrhoea, weight loss, and anaemia.

### 2.3. Serum Markers

We included in the study the analysis of four different serum markers that were chosen for their potential role in discriminating a complicated form of CD. In particular, CgA, a neuroendocrine secretory protein, is a marker of neuroendocrine cell proliferation. It has already been shown that patients with RCD have an increased number of neuroendocrine cells within the duodenal mucosa [18]. Instead, β2-microglobuline and lactate dehydrogenase (LDH) were assessed as potential markers of the presence of aberrant lymphocytes and of increased apoptosis, respectively [19,20]. Albumin was assessed as a marker of malabsorption. Serum CgA was determined by solid-phase two-site immunoradiometric assay (CIS Bio International, Gif-sur-Yvette, France). Serum β2-microglobuline was determined by mass spectrometric immunoassay (Abcam, ELISA kit, UK). LDH and albumin were determined by fluorometric assay (Abcam assay kit, Cambridge, UK) and colorimetric assay (Abcam assay kit, Cambridge, UK), respectively. For each patient enrolled, all the aforementioned serum markers were analysed at baseline and after a median period of 13 months of follow-up.

### 2.4. Statistical Analysis

Continuous data were described with median and 25–75th percentiles (except for age, which was reported as mean and standard deviation (SD)) and categorical data as counts and percentages. Biomarker values were compared between groups with the Mann–Whitney U-test and within groups with the Wilcoxon sign-rank test. Receiver Operator Characteristic (ROC) curve analysis was performed to identify the optimal cut-off maximizing sensitivity and specificity. A 2-sided *p*-value < 0.05 was considered statistically significant. Given the exploratory, proof-of-concept, nature of the study, no multiple comparisons *p*-value adjustment are presented.

The STATA software (release 16, StataCorp, College Station, TX, USA) was used for computation.

## 3. Results

With the aim of identifying serum markers that can predict refractoriness in patients with CD, serum levels of chromogranin A (CgA), β2-microglobuline, lactate dehydrogenase (LDH), and albumin were measured at baseline in 72 patients affected by CD, and divided into three groups: patients with RCD (either type 1 or 2), patients with uncomplicated CD before GFD, and the same uncomplicated CD patients in whom the response to GFD was ascertained histologically after at least 12 months (defined from here onwards as treated CD (TCD)). All RCD patients were following a strict GFD, according to a clinically validated score of adherence [16]. In all RCD patients, jejunoileitis and EATL were excluded at the time of assessment. Serologic follow-up was also performed after at least 12 months. Laboratory data are summarized in Table 1.

### 3.1. Serum Levels of CgA

**Figure 1:**
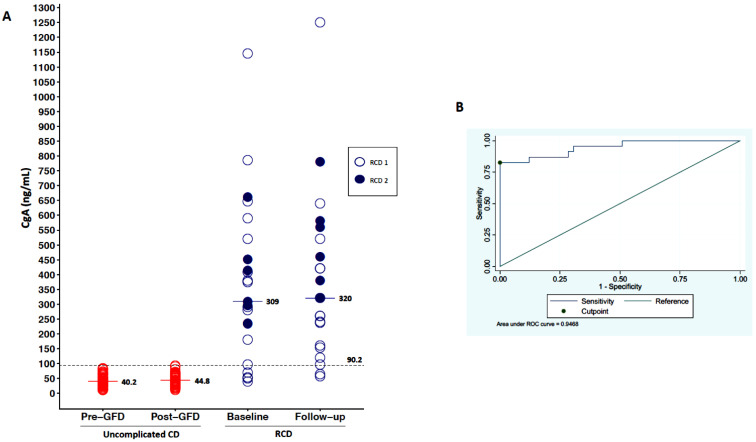
Variation of CgA levels. (**A**) shows the variability of CgA levels in the three groups of patients (uncomplicated CD, RCD type 1, RCD type 2) at baseline and at follow-up. Serum levels of CgA at baseline were higher in patients with RCD in comparison to uncomplicated CD patients before-GFD. (**B**) is an ROC curve that shows an empirical cut-off point of CgA (90.2 ng/mL), which is able to differentiate uncomplicated CD vs. RCD patients, with a sensitivity of 83% and a specificity of 100%.

As shown in Figure 1A, serum levels of CgA were higher in patients with RCD (median 309 ng/mL, IQR 235–450) in comparison to uncomplicated CD patients before a GFD (median 40 ng/mL, IQR 26–55; *p* < 0.001). When we stratified RCD patients according to the sub-type, we observed no significant difference between type 1 and type 2 (Table 1). An empirical cut-off point of CgA, able to differentiate uncomplicated CD vs. RCD patients, was estimated as 90.2 ng/mL, with a sensitivity of 83% and a specificity of 100% (see the ROC curve in Figure 1B). CgA was then assessed at follow-up (median time of 13 months), having a median of 45 ng/mL (IQR 32–60) in TCD, while had a median of 250 ng/mL (IQR 137–420) in RCD type 1 and 460 ng/mL (IQR 320–580) in RCD type 2 patients.

At the last follow-up, three patients developed an EATL. Both baseline and follow-up CgA were not able to predict the evolution of RCD into EATL, as this marker was always elevated in all cases.

### 3.2. Serum Levels of β2-Microglobuline

**Figure 2:**
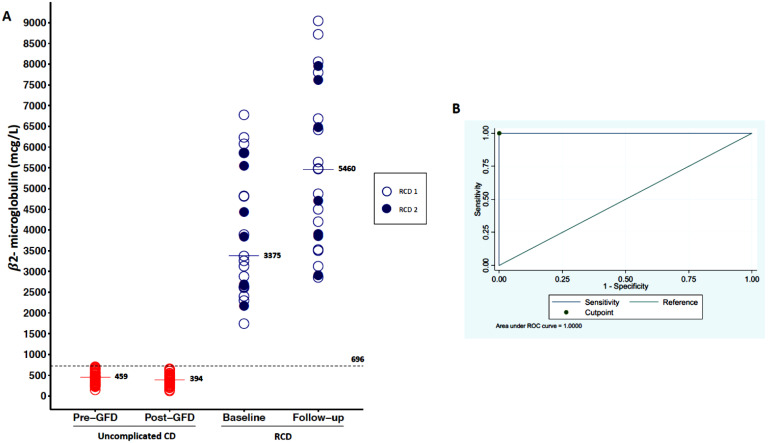
Variation of β2-microglubine levels. (**A**) shows the variability of β2-microglubine levels in the three groups of patients (uncomplicated CD, RCD type 1, RCD type 2) at baseline and at follow-up. Serum levels of β2-microglubine at baseline were higher in patients with RCD in comparison to uncomplicated CD before a GFD. (**B**) is an ROC curve that show an empirical cut-off point of β2-microglubine (696 mcg/L), which is able to differentiate uncomplicated CD vs. RCD patients with a sensitivity of 100% and a specificity of 100%.

As shown in Figure 2A, serum levels of β2-microglubine were higher in patients with RCD (median 3375 mcg/L, IQR 2626–5542) in comparison to uncomplicated CD before a GFD (median 459 mcg/L, IQR 322–552; *p* < 0.001). When we stratified RCD patients according to the type, we observed no significant difference between type 1 and type 2 (Table 1). An empirical cut-off point of β2-microglubine, able to differentiate uncomplicated CD vs. RCD patients, was estimated as 696 mcg/l, with a sensitivity of 100% and a specificity of 100% (see ROC curve in Figure 2B). β2-microglubine was then assessed at follow-up (median of 13 months), with a median of 394 mcg/L (IQR 302–470) in TCD, while it had a median of 5470 ng/mL (IQR 3862–7240) in RCD type 1 and 4700 ng/mL (IQR 3850–7620) in RCD type 2.

Both baseline and follow-up β2-microglubine were not able to predict the evolution of RCD into EATL, as this marker was always elevated in all cases.

### 3.3. Serum Levels of LDH and Albumin

**Figure 3:**
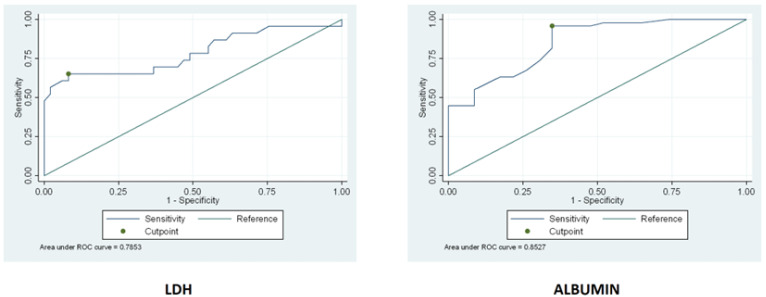
The figure below represents the ROC curves of LDH and ALBUMIN. A cut-off point was calculated for both these biomarkers in order to predict refractoriness.

Serum levels of LDH and albumin were assessed in uncomplicated CD patients before a GFD and in RCD patients. LDH was significantly (*p*-value < 0.001) higher in RCD (median 220 mUI/mL, IQR 168–317) than uncomplicated CD before GFD (median 167, IQR 146–192), with no significant difference between RCD type 1 and type 2 patients (Table 1). Albumin, on the other hand, was significantly lower (*p*-value < 0.001) in RCD patients (median 3.2 g/L IQR 2.8–4.0) than uncomplicated CD patients before a GFD (median 4.3 g/dL, IQR 3.8–5.2), with no significant difference between RCD type 1 and type 2 patients (Table 1). Hence, a cut-off point was calculated for both these biomarkers in order to predict refractoriness. For LDH, the cut-off was 206 mUI/mL with a sensitivity of 65% and a specificity of 92%. For albumin, the cut-off was 3.3 g/dL with a sensitivity of 96% and a specificity of 65% (see the ROC curves in Figure 3). LDH and albumin proved not to be useful markers of refractoriness, having inferior sensitivity and specificity compared to CgA and β2-microglubine.

## 4. Discussion

We have herein shown that CgA and β2-microglobuline are accurate markers for discriminating uncomplicated CD from RCD. In CD patients who manifest signs or symptoms of malabsorption or other gastrointestinal complaints after a period of well-being with a strict GFD, nonresponsive CD should be suspected. Under this umbrella term, several different clinical conditions can be encompassed, including inadvertent gluten intake, gastrointestinal disorders concomitant to CD (e.g., irritable bowel syndrome, lactose intolerance, small intestinal bacterial overgrowth, etc.), and true RCD; a correct differential diagnosis is crucial in this setting. CD serology is of little usefulness, as it is negative in most cases, and serum biomarkers reflecting the enterocyte mass are not available in routine clinical practice yet [21]. Thus, it would be useful to have noninvasive tests able to identify those patients who should undergo upper gastrointestinal endoscopy with the collection of duodenal biopsies because of the high suspicion of being affected by RCD.

Starting from the rationale of a previous study of ours, which demonstrated a higher proportion of CgA-positive cells in RCD patients’ duodenal mucosa in comparison to uncomplicated CD [18], we here detected serum levels of CgA in patients followed at our centre affected by RCD and uncomplicated CD. We have also determined serum β2-microglobuline, LDH, and albumin. CgA and β2-microglobuline resulted higher in patients with RCD compared to uncomplicated CD, both at baseline and follow-up. Specifically, CgA and β2-microglobuline showed high sensitivity and specificity to differentiate RCD patients from all uncomplicated CD patients, either treated or untreated. Both markers, moreover, have been shown to remain stable over time, at least after a median time of 13 months. LDH and albumin showed not to be useful biomarkers of refractoriness, having inferior sensitivity and specificity. With the same specificity, β2-microglobuline has therefore shown to be more sensitive than CgA in predicting the possible refractoriness of CD. However, there are a great number of physio-pathological conditions that can lead to an increase in β2-microglobuline values, such as lymphoproliferative disorders, neoplastic diseases, infectious diseases, chronic kidney failure, and any inflammatory conditions [22]. We, therefore, believe that CgA is preferable in clinical practice as a refractoriness marker. Indeed, even CgA serum levels may be increased in other conditions, such as autoimmune atrophic gastritis or *Helicobacter pylori* infection, which can be easily ruled out through highly accurate noninvasive markers [23,24]. Indeed, we also recommend testing for CgA only patients who are not on proton pump inhibitors, or at least after a 2-week wash-out [25]. Moreover, even if increased duodenal CgA-producing cells are found in the duodenum of patients with irritable bowel syndrome [26], plasma CgA levels are normal [27]. Finally, plasma CgA was found to be increased in inflammatory bowel disease [28], which, however, can be ruled out by means of the clinical picture (e.g., bloody diarrhea, abdominal pain in the lower quadrants), radiologic (e.g., ultrasound, computer tomography scan, magnetic resonance), and endoscopic (e.g., colonoscopy) examinations. In any case, if a patient with a suspected RCD presented with the aforementioned conditions, β2-microglobuline could be used as the primary marker.

This study has some limitations that should be mentioned. First, the sample size is rather small, and patients were enrolled in a tertiary referral center for the management of patients with CD and its complications. In fact, the number of included patients with RCD is far higher compared to that of patients with CD, when considering the prevalence of these two conditions. Indeed, this markedly increases the pre-test probability of diagnosing refractoriness. Hence, to corroborate our results, a prospective validation study is needed to assess the accuracy of these serum markers in different control groups (i.e., patients with other gastrointestinal conditions, such as common variable immune deficiency-associated enteropathy, other atrophic enteropathies, irritable bowel syndrome) and in the general population or a primary care setting. In addition, the lack of other control groups led to a 100% accuracy of β2-microglobuline, which is indeed explained by the high ceiling effect. Adherence to a GFD was assessed through a validated score which, however, may have some limitations. Finally, only three patients eventually developed an EATL at the last follow-up, and hence we cannot draw any firm conclusion in these patients. Nonetheless, we have herein found a potential, inexpensive serum marker—CgA—able to address the diagnosis of RCD, the diagnosis of which must be confirmed by phenotypical characterization through flow-cytometry of the isolated IELs and by assessment of T-cell receptors β and/or γ rearrangement through RT-PCR [11,29].

## 5. Conclusions

In conclusion, given that RCD can only be diagnosed by means of histology, we propose the use of either CgA or β2-microglobuline as noninvasive markers of RCD, especially to differentiate this condition from other forms of nonresponsive CD characterized by the lack of villous atrophy. By doing so, we can establish the need for searching for aberrant IELs and T-cell receptors β and/or γ rearrangement before scheduling an upper endoscopic examination. A prospective validation study in other settings is needed to confirm our preliminary results.

## Figures and Tables

**Table 1:** Biomarkers values (median and IQR) at baseline and follow-up.

Biomarker	Type	Baseline	Follow-Up	*p*-Value ^3^	Change
CgA (ng/mL)	Uncomplicat-ed CD RCD	40 (26–55) 309 (180–520)	45 (32–60) 320 (160–520)	0.001 0.119	4 (−0.6–7) 63 (13–112)
	*p*-value ^1^	<0.001	<0.001	/	<0.001
	RCD 1 RCD 2	332 (83–555) 309 (235–450)	250 (137–420) 460 (320–580)	0.632 0.016	41 (−140–72) 118 (85–147)
	*p*-value ^2^	0.285	0.196	/	0.005
β2-microglobuline (mcg/L)	Uncomplicat-ed CD RCD	459 (322–552) 3375 (2626–5542)	394 (302–470) 5460 (3850–7620)	<0.001 <0.001	−24 (−75–−2) 1620 (1020–2054)
	*p*-value ^1^	<0.001	<0.001	/	<0.001
	RCD 1 RCD 2	3321 (2613–5340) 3840 (2655–5542)	5740 (3862–7240) 4700 (3850–7620)	<0.001 0.015	1685 (1068–2172) 1211 (860–2054)
	*p*-value ^2^	0.487	0.456	/	0.431
LDH (mUI/mL)	Uncomplicat-ed CD RCD	167 (146–192) 220 (168–317)	NA NA	/ /	NA NA
	*p*-value ^1^	<0.001	/		
	RCD 1 RCD 2	218 (163–312) 289 (179–395)	NA NA	/ /	NA NA
	*p*-value ^2^	0.131	/		
Albumin (g/dL)	Uncomlicate-d CD RCD	4.3 (3.8–5.2) 3.2 (2.8–4.0)	NA NA	/ /	NA NA
	*p*-value ^1^	<0.001	/		
	RCD 1 RCD 2	3.2 (2.8–4.0) 3.1 (2.4–3.8)	NA NA	/ /	NA NA
	*p*-value ^2^	0.360	/		

Legend: ^1^ comparison between types; ^2^ comparison between sub-types; ^3^ comparison within types or subtypes. NA = not assessed.

## Data Availability

All data have been included in the present manuscript.
